# Giant fibrolipoma mimicking abdominal lipodystrophy

**DOI:** 10.4103/0970-0358.41127

**Published:** 2008

**Authors:** Yusuf Kenan Coban, Ayhan Coskun

**Affiliations:** Sütcü Imam Üniversity, Medical Faculty Plastic Surgery Department, Kahramanmaras, Turkey; 1 Obstetric and Gynecology Department, Kahramanmaras, Turkey

Dear Sir,

Fibrolipomas belong to the family of fat-containing lesions and are benign tumor variants of lipomas characterized by the presence of adipose tissue and abundant amounts of fibrous tissues. They are well-separated from the surrounding tissues and usually occur in adults.[1] These lesions may have a broad base or may be pedinculated.[2,3] Fibrolipomas are one of the giant lipomatous tumors (defined as being greater than 5 cm in diameter), which have been reported to be seen in the esophagus, intestinal mesentery, pancreas and the parapharyngeal region.[4–7] Owing to the peculiarity of this condition and the difficulties encountered in its diagnosis and treatment, we report here a case of a giant fibrolipoma in the abdomen.

**Figure 1:**
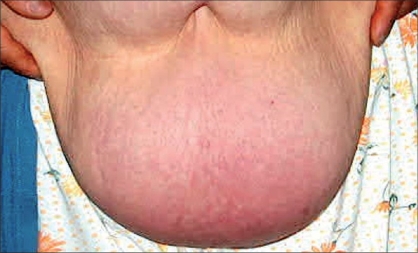
The clinical picture of the mass

**Figure 2:**
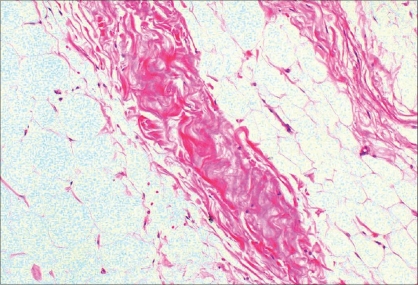
Mature adipocytes within the fibrohyaline bands are seen (Hematoxylen andeosin 200× magnification)

**Figure 3:**
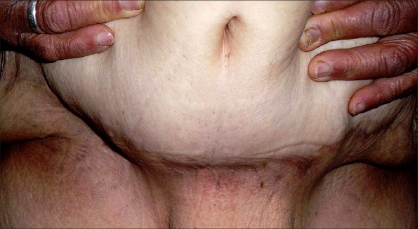
Late postoperative photo showing the low abdominal scar

A 62-year-old female presented with a giant mass in the abdominal wall [Figure 1]. The first visual impression resembled a flask abdomen with severe lipodystrophy. Pre-operative evaluation using ultrasonography showed a very large subcutaneous mass with intact anterior abdominal integrity except for a moderate rectus muscle diastasis. Clinical examination revealed that the mass was strictly attached to the abdominal wall with a wide pedicle and there was no tenderness over it. The patient's main complaint was cosmetic. The patient had intermittent cardiac failure and had been undergoing treatment for five years; otherwise, there was no associated medical problem. The mass of 12 × 10 × 3 cm was excised under general anesthesia [Figure 1]. The surgical approach consisted of excising the mass with a transverse low abdominoplasty incision. No rectus plication or undermining was done. There was no wound healing problem in the postoperative period. The microscopic evaluation of the mass (weighing 1517 g) revealed an admixture of mature adipocytes and fibrous connective tissue [Figure 2]. Pathological examinations revealed that the tumor was composed of spindle cell-like fibroblasts and mature adipocytes. Immunohistochemical examinations revealed that the tumor was negative for desmin and alpha-smooth muscle actin. Based on these findings, a diagnosis of fibrolipoma was made. One year after the surgery, both the local and the general condition of the patient were good and there were no signs of recurrence [Figure 3]. Incidentally another interesting clinical finding was also noted; the clinical cardiac symptoms also improved after the surgery.

Neural fibrolipoma is a benign tumor comprised of hypertrophied, fibrofatty tissue with intermixed nerve tissue and fibrolipomatous hamartoma of the nerve usually seen in infants and children.[8] Although these lesions are often asymptomatic, they may cause discomfort when they become large. As far as our literature review goes, there are no similar reports for fibrolipomas such as the one presented here. The main feature of this fibrolipoma is its resemblance of abdominal lipodystrophy. A subcutaneous fibrolipoma has been described by Kajihara *et al*. in the back region.[9] For a lipoma to be referred to as “giant”, the lesion should be at least 10 cm in diameter or weigh a minimum of 1000 g. These criteria were also met in this case, so the lesion may also be called a giant fibrolipoma. It is not clear as to what mechanisms, excluding trauma, trigger these giant fibrolipomas and the differences of these lesions from other types of lipomas are only based on histopathologic findings.[10] In the future, cellular and subcellular research will perhaps determine physiopathologic mechanisms underlying the progress of such lipoma-like lesions.
